# Analysis of Efficacy and Toxicity Profile of First-Line Sunitinib or Pazopanib in Metastatic Clear Cell Renal Cell Carcinoma in the Brazilian Population

**DOI:** 10.1200/JGO.18.00073

**Published:** 2018-09-10

**Authors:** Pedro Isaacsson Velho, Mirella Nardo, Manoel Carlos Leonardi de Azevedo Souza, Renata R.C. Colombo Bonadio, Guilherme Nader Marta, David Q.B. Muniz, Diogo Assed Bastos, Carlos Dzik

**Affiliations:** **Pedro Isaacsson Velho**, Johns Hopkins University, Baltimore, MD, and Hospital Moinhos de Vento, Porto Alegre; and **Mirella Nardo**, **Manoel Carlos Leonardi de Azevedo Souza**, **Renata R.C. Colombo Bonadio**, **Guilherme Nader Marta**, **David Q.B. Muniz**, **Diogo Assed Bastos**, and **Carlos Dzik**, Instituto do Cancer do Estado de Sao Paulo, São Paulo, Brazil.

## Abstract

**Purpose:**

Sunitinib and pazopanib are multitargeted tyrosine kinase inhibitors (TKIs) that act against vascular endothelial growth factor receptors and are standard first-line treatment options for metastatic clear cell renal cell carcinoma (ccRCC). The Brazilian public health system diverges from the randomized clinical trials in the availability of first and subsequent lines of treatment and in clinical and demographic characteristics of patients. Therefore, it is essential to describe the history of advanced ccRCC during and after TKI treatment in this population.

**Methods:**

We performed a retrospective analysis of patients with advanced ccRCC treated with a first-line TKI (either sunitinib or pazopanib) between February 2009 and March 2017 in a single academic Brazilian cancer center (Instituto do Câncer do Estado de São Paulo).

**Results:**

Of the 222 patients, 109 were treated with sunitinib and 113 with pazopanib. The median duration of treatment and overall survival (OS) were 6.4 and 15.2 months for sunitinib and 6.7 and 14.2 months for pazopanib, respectively. Discontinuation of treatment occurred secondarily to progressive disease or death in 64.2% of patients using sunitinib and in 54.8% of patients using pazopanib. Adverse events were responsible for discontinuation of treatment in 28.4% of patients in the sunitinib group and in 22.1% in the pazopanib group. According to Memorial Sloan-Kettering Cancer Center risk categories, the OS was 32.9 months, 15.9 months, and 8.1 months for low risk, intermediate risk, and poor risk, respectively (hazard ratio, 1.72; 95% CI, 1.13 to 2.26; *P* < .001).

**Conclusion:**

The use of TKI inhibitors as first-line treatment of metastatic RCC is effective and feasible in the Brazilian public health. However, the median OS of our population is considerably lower compared with the prospective trials that evaluated the same drugs.

## INTRODUCTION

The treatment of advanced clear cell renal cell carcinoma (ccRCC) has changed in recent years since the benefit of targeted therapies and immunotherapy has been demonstrated.^1^ Both pazopanib and sunitinib are multitargeted tyrosine kinase inhibitors (TKIs) that act against vascular endothelial growth factor receptors and since 2006 are standard first-line treatment options for metastatic ccRCC.^2,3^ The combination of bevacizumab and interferon alfa and the mammalian target of rapamycin inhibitor temsirolimus (for poor-risk patients) also are options for first-line treatment.^4,5^ Recently, two combinations of drugs, nivolumab plus ipilimumab^6^ and atezolizumab plus bevacizumab,^7^ also demonstrated impressive results in phase III trials in patients with previously untreated advanced ccRCC and might become first-line treatment options as well.

Because of the efficacy, good tolerability, and convenience of the oral agents, these two TKIs, either sunitinib or pazopanib, are largely used in the first-line setting, especially for patients in favorable- and intermediate-risk groups. In a direct comparison between these two drugs, pazopanib demonstrated noninferiority to sunitinib in terms of progression-free survival (PFS) and overall survival (OS).^8,9^ These two drugs have different patterns of adverse effects (AEs), with sunitinib being more associated with fatigue, hand-foot syndrome, and hematologic toxicity and pazopanib being more associated with abnormalities in liver function tests (total bilirubin, AST, ALT, and alkaline phosphatase).^8^ Despite the differences in AEs and the patient preference for pazopanib over sunitinib,^10^ both medications have similar chances of discontinuation because of toxicities (20% *v* 24% for sunitinib and pazopanib, respectively).^8^

Unfortunately, in the Brazilian public health system, not all institutions are able to provide access to pazopanib or sunitinib for patients with metastatic ccRCC. Also, outside clinical trials, patients in Brazilian public health have limited access to second- and third-line therapies, which may negatively affect the OS of this population, because it has been demonstrated that patients who are able to receive more lines of systemic therapy live longer.^11^ Moreover, two recent trials with nivolumab and cabozantinib demonstrated improvement in OS compared with everolimus.^12,13^ The scenario of ccRCC in Brazilian public health diverges importantly from the randomized clinical trials, not only in the availability of first and subsequent lines of treatment but also in clinical and demographic characteristics of the patients. Considering that, it is essential to describe and recognize the history of advanced ccRCC during and after TKI treatment, including the impact of prognostic factor models, in Brazilian patients treated in the public health system. This study aims to evaluate the real-life outcomes of patients with metastatic ccRCC who had access to first-line therapies and were treated with either sunitinib or pazopanib in a single cancer center in Brazil, evaluating the effect of the prognostic risk groups, toxicities, and the outcomes of these patients.

## METHODS

### Study Design and Participants

We performed a retrospective analysis of patients with advanced ccRCC treated with a first-line TKI (either sunitinib or pazopanib) between February 2009 and March 2017 in a single academic Brazilian cancer center (Instituto do Câncer do Estado de São Paulo). From February 2009 until September 2013, sunitinib was the standard first-line TKI available in this institution for metastatic ccRCC. From September 2013 until the end of the study in March 2017, pazopanib was the first-line treatment available. Medical records were reviewed to access outcomes and clinical and demographic characteristics of patients.

Patients were categorized into risk groups according to five risk factors on the basis of the Memorial Sloan Kettering Cancer Center (MSKCC) prognostic factors model: Karnofsky performance score (KPS) < 80, time from diagnosis to treatment < 1 year, hemoglobin levels less than the lower limit of normal, corrected serum calcium > 10 mg/dL, and serum lactic dehydrogenase level > 1.5 times the upper limit of normal.^10^ Patients with zero risk factors are considered as favorable risk, patients with one or two risk factors are considered intermediate risk, and patients with three or more risk factors are in the poor-risk group.

Data were also collected to evaluate if the treated patients presented characteristics considered as exclusion criteria in the phase III trials that evaluated vascular endothelial growth factor receptor TKIs in previously untreated advanced ccRCC.^2,3^ These criteria included KPS < 70, inadequate organic function (hematologic, renal, hepatic, coagulation, or cardiac), CNS metastasis, and clinically significant cardiovascular events during the preceding 12 months. This analysis was done to compare the real-life patients with those of the randomized studies. Local research ethics committees approved the study.

### Treatment

Sunitinib was given orally at a dose of 50 mg per day on a schedule of 4 weeks on and 2 weeks off treatment or, alternatively, at 37.5 mg once per day continuously. Pazopanib was given orally at 800 mg once per day. Dose reductions of pazopanib for 600 mg or 400 mg once per day were permitted if necessary. Dose reductions and reasons for dose reductions or drug discontinuation were registered. Patients were included if they received at least one dose of sunitinib or pazopanib.

### Statistical Analysis

The relative and absolute frequencies of clinical and demographic characteristics were tabulated. Unpaired *t* test was used to compare continuous variables between treatment groups and χ^2^ test was used for categorical variables. A descriptive comparison was done to evaluate toxicities, the proportion of dose reductions, and the proportion of treatment interruptions in the sunitinib versus the pazopanib group. We evaluated the duration of treatment and OS according to the treatment received (either sunitinib or pazopanib) and stratifying according to the risk group (favorable-, intermediate-, or poor-risk group).

Duration of treatment was considered the time from initiation of TKI until TKI discontinuation because of documented clinical or radiologic progression, unacceptable toxicity, or death for any cause, whichever occurred first. OS was defined as the time from initiation of TKI until death for any cause. Patients without these events were censored at the time of last follow-up. Survival analyses were performed using the Kaplan-Meyer method. The difference between survival curves was evaluated with the log-rank test. Univariable and multivariable analyses were performed using the Cox proportional model to evaluate prognostic factors. Variables with a *P* < .1 in the univariable analysis were included in the multivariable analysis. Statistical analyses were performed using Stata software, version 14 (StataCorp, College Station, TX). A *P* value < .05 was considered statistically significant.

## RESULTS

### Patients/Demographic Characteristics

Between February 2009 and March 2017, 222 patients who were diagnosed with metastatic ccRCC at Instituto do Cancer do Estado de Sao Paulo received either sunitinib (n = 109) or pazopanib (n = 113) as first-line therapy. Demographic and clinical characteristics at baseline were balanced between the treatment groups. Demographic, clinical, and pathologic characteristics of the patient cohort are shown in Table 1. Overall, patients had a median age of 60 years (range, 19 to 86 years), and 65% were men. The majority of patients had prior nephrectomy (72%), and 65% had a KPS > 80. Patients classified as poor risk at diagnosis in the MSKCC prognostic model were 29% in the overall population and were slightly different between the two groups (25.6% in the sunitinib group and 32.7% in the pazopanib group; *P* = .121). Lung metastasis was present in 75% of patients in both groups and was the most common site of metastasis, followed by lymph nodes (62.1%), bone (39%), and liver (20%).

**Table 1:**
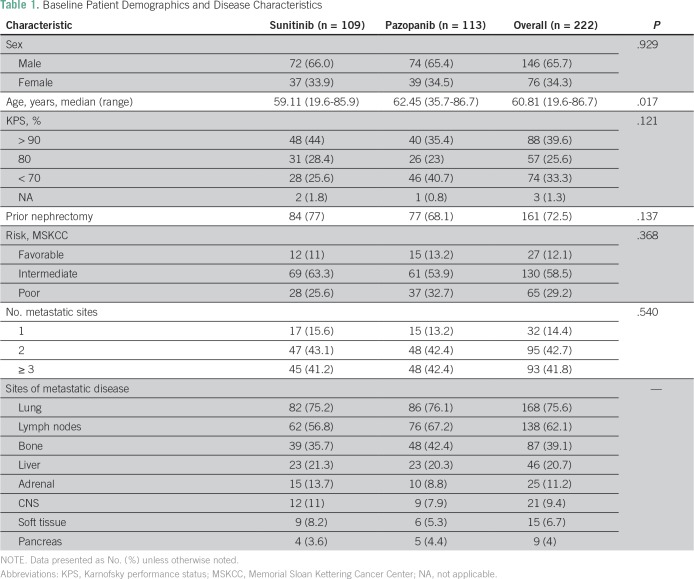
Baseline Patient Demographics and Disease Characteristics

### Toxicities, Duration of Therapy, and Efficacy

**Fig A1:**
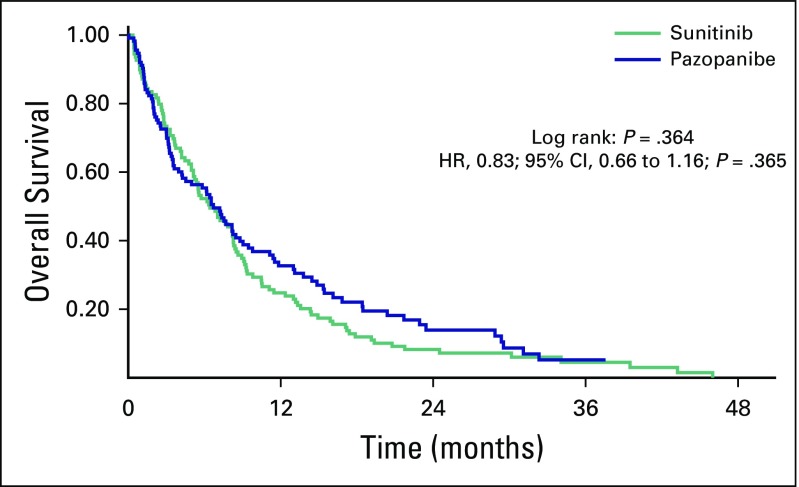
Median of duration of treatment for the two groups. HR, hazard ratio.

The duration of treatment between the two groups was similar: 6.7 months for pazopanib and 6.4 months for sunitinib (hazard ratio [HR], 0.83; 95% CI, 0.66 to 1.16; *P* = .364; Appendix Fig A1). Of the patients with available data for toxicity and disease progression (84.6%), the major reason for discontinuation of treatment was progressive disease or death, corresponding to 64.2% and 54.8% of patients who received sunitinib or pazopanib, respectively. AEs were responsible for treatment discontinuation in 28.4% of patients receiving sunitinib and 22.1% of patients receiving pazopanib.

The description of the AEs is listed in Appendix Table A1. Fatigue, diarrhea, and nausea/vomiting were the most common toxicities associated with therapy in both groups, with similar incidence between them. Hand-foot syndrome, hypertension, and thrombocytopenia occurred more commonly in the sunitinib group, and hepatotoxicity was more common in the pazopanib group.

**Fig 1:**
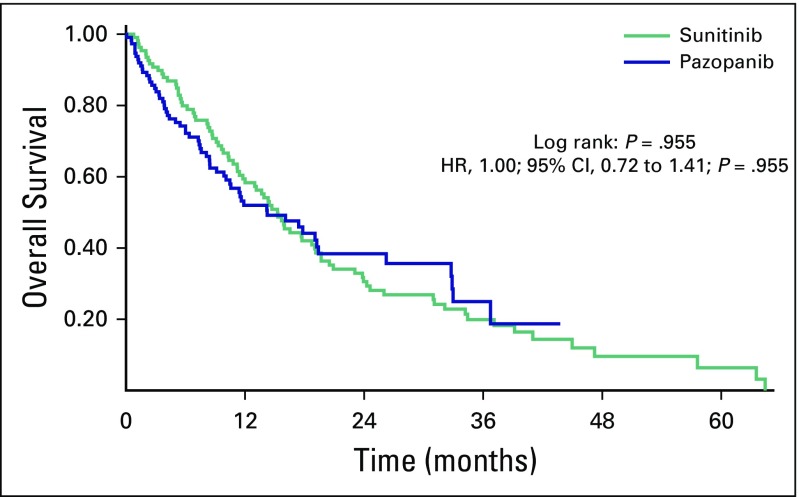
Median overall survival for patients who received sunitinib or pazopanib. HR, hazard ratio.

**Fig 2:**
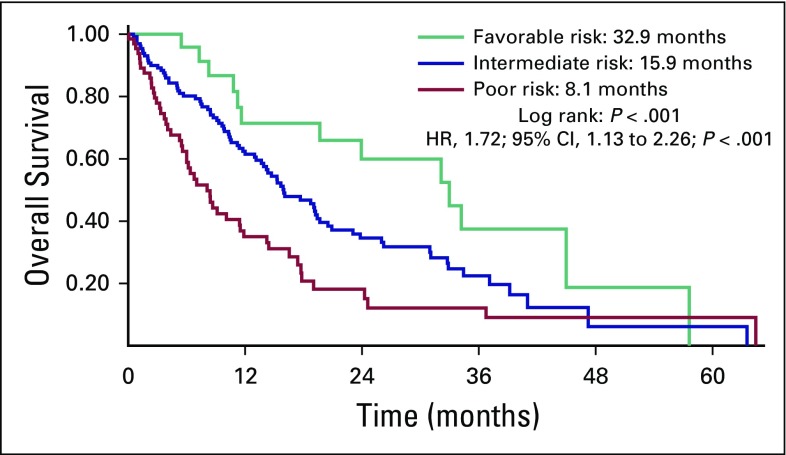
Median overall survival for the different Memorial Sloan Kettering Cancer Center prognostic groups. HR, hazard ratio.

**Fig 3:**
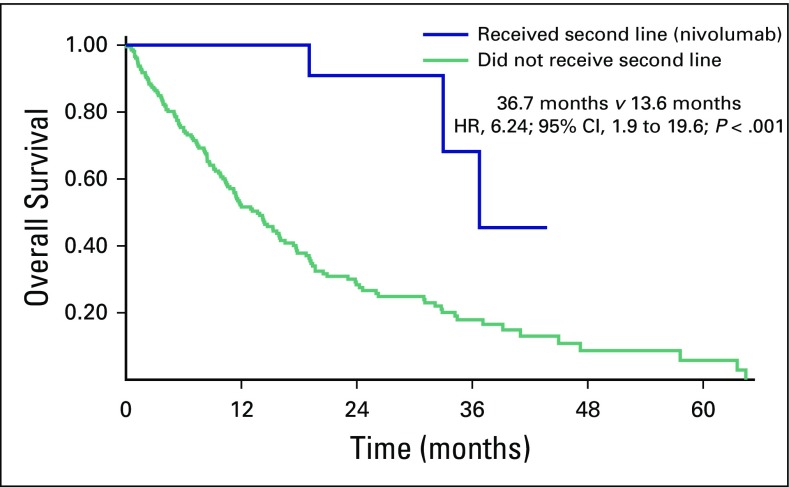
Overall survival of patients who received second-line treatment with nivolumab and patients who did not. HR, hazard ratio.

OS was 15.2 months and 14.2 months for sunitinib and pazopanib, respectively (HR, 1.00; 95% CI, 0.72 to 1.41; *P* = .955; Fig 1). Among patients with the three MSKCC risk categories, the OS was 32.9 months, 15.9 months, and 8.1 months for low risk, intermediate risk, and poor risk, respectively (HR, 1.72; 95% CI, 1.13 to 2.26; *P* < .001; Fig 2). An exploratory analysis of the 14 patients in this cohort who were treated under the compassionate use program of nivolumab after disease progression on sunitinib or pazopanib showed that these patients had better OS compared with patients who did not have access to a second-line therapy (36.7 months *v* 13.6 months; HR, 6.24; 95% CI, 1.9 to 19.6; *P* < .001; Fig 3).

**Fig 4:**
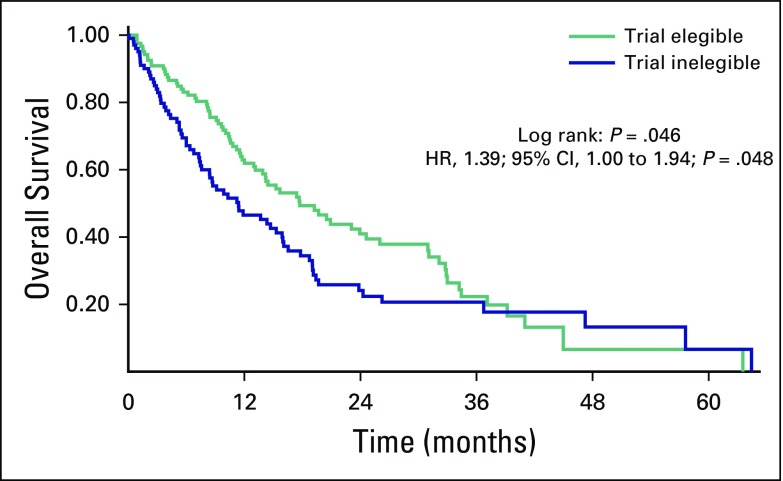
Median overall survival for patient who would be eligible for clinical trials and patients who would not be eligible for clinical trials. HR, hazard ratio.

Our study also analyzed differences in median OS between patients who would be eligible for clinical trials and patients who would not be eligible for clinical trials (KPS < 70, CNS metastasis, Hb < 9 g/dL, or any cardiovascular serious event, such as myocardial infarction unstable angina, or pulmonary embolism, in the last 12 months). Applying these criteria, of the 222 patients, 101 (45.5%) would be ineligible for the vast majority of phase III clinical trials. The median OS of these patients was 11.4 months, versus 17.7 months for patients who would be eligible for clinical trials (HR, 1.39; 95% CI, 1.00 to 1.94; *P* = .048; Fig 4). Univariable and multivariable analyses were done evaluating different prognostic factors, such as age > 60 years, MSKCC risk group, neutrophil-lymphocyte ratio (NLR) < 3 versus ≥ 3, number of metastatic sites, presence of CNS metastases, eligibility for phase III clinical trials (yes or no), and treatment group (sunitinib *v* pazopanib). Of these, three criteria demonstrated to be prognostic in the univariable analysis in our group of patients: MSKCC risk group (*P* < .001), NLR (*P* = .001), and trial eligibility (*P* = .048). Two of these criteria also showed prognostic importance in the multivariable analysis: MSKCC risk group (*P* = .003) and NLR (*P* = .016), corroborating previous prospective data (Table A2).

## DISCUSSION

In the past decade, treatment options for metastatic ccRCC have been expanded. Until 10 years ago, interferon alfa was the mainstay of treatment of this disease. Although this therapy provides response rates of 10% to 12%, the level of toxicity is high, and there is no benefit in OS.^14^ During the year 2000, when the US Food and Drug Administration approved sunitinib and pazopanib for metastatic ccRCC, patients started benefiting from these medications on the basis of their improvement in response rate, PFS, and even in OS, especially in those who are exposed to multiple lines of therapy.^11,15-17^

Our study showed that patients with metastatic ccRCC who received treatment in the Brazilian public health system have worse OS compared with the phase III trial in the same population.^2,3,9^ There are several reasons for this result. First, the baseline characteristics of our patients, such as KPS and MSKCC prognostic classification, are worse than in the phase III trials in the same setting, which therefore could explain the worse OS in our study. In the pivotal Pazopanib Versus Sunitinib in Metastatic Renal Cell Carcinoma (COMPARZ) study,^8^ patients with a KPS < 70 were not eligible, and in our cohort, 33.3% of patients had a KPS ≤ 70. In addition, patients who had fulfilled criteria as a poor-risk MSKCC prognostic group^18^ were approximately 10% in the COMPARZ and > 30% in our cohort (Table 2). It is important to highlight that the MSKCC prognostic classification, when applied in our patients, showed clear OS difference among the three prognostic groups, validating this classification in the Brazilian population. Another relevant characteristic of our patients that also elucidates the poor prognosis of our patients is the presence of brain metastasis (9.4%). Prospective data of a TKI expanded-access trial showed that patients with CNS metastasis secondary to ccRCC have poor prognosis, with a median OS of 9.2 months, compared with 18.4 months in the overall population.^19^

**Table 2:**
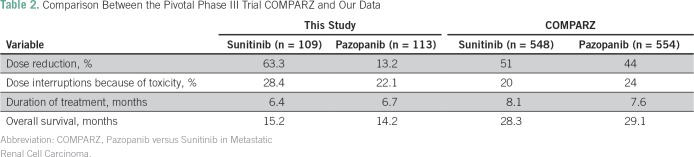
Comparison Between the Pivotal Phase III Trial COMPARZ and Our Data

Several studies showed that up to 60% of real-life patients would be ineligible for clinical trials because of inability to meet the strict eligibility criteria.^20^ In our cohort, 45.5% of patients would not be eligible for clinical trials because of multiple factors, such as low KPS, CNS metastasis, Hb < 9 g/dL, and previous cardiovascular conditions within the past 12 months (such as myocardial infarction, unstable angina, class III or IV congestive heart failure, and pulmonary embolism). The median OS of our cohort improves when we evaluate only patients who would be eligible in phase III clinical trials (17.7 months) compared with patients who would be excluded from them (11.4 months). This demonstrates that more strict criteria might be followed to offer systemic therapy for patients with baseline poor prognosis, because certainly some of them do not benefit from any systemic therapy. Perhaps best supportive care would be more appropriate for some of them.

Also, despite a similar duration of treatment between the COMPARZ (8.1 months in sunitinib arm and 7.6 months in pazopanib arm) and our study (6.4 months for sunitinib and 6.7 months for pazopanib), a significant difference in median OS was seen. The updated OS for the phase III study was 28.3 months in the pazopanib group and 29.1 months in the sunitinib group, whereas in our cohort it was 14.2 months and 15.2 months, respectively. Certainly, the differences between the baseline characteristics of our patients (inferior KPS, more poor-risk patients, and the presence of brain metastasis) could, in part, explain the difference in survival; however, these are not the only possible reasons. Retrospective data comparing the early (2006 to 2009) and late (2010 to 2012) targeted therapy eras in the treatment of ccRCC showed that the median OS was significantly longer in the late than in the early targeted-therapy era, suggesting that better survival had resulted from greater knowledge in using TKIs by the treating physicians. We believe this should be considered in our analysis as well.

In the Brazilian public health system, there is no standard systemic treatment option, outside of a clinical trial, for patients with metastatic ccRCC who have progressed to a TKI as first-line treatment. In the seminal study, after disease progression on pazopanib or sunitinib, other antiangiogenesis agents or mammalian target of rapamycin inhibitors were administered in > 50% of patients.^9^ Although not reported, probably many of them received nivolumab and/or cabozantinib as well, both of which demonstrated OS benefit in metastatic ccRCC.^12,13^ In our cohort, 14 patients received nivolumab after disease progression while receiving TKIs. This subgroup of patients had a better median OS than the patients who did not receive any treatment after disease progression (36.7 months; HR, 6.24; 95% CI, 1.98 to 19.65). Several criticisms could be made to this analysis, such as the small number of patients and selection bias; however, it is reasonable to consider that some of these patients survived longer because of the second-line treatment. This is reinforced by the data showing that offering multiple lines of treatment to real-life patients with ccRCC increases median OS,^17^ suggesting this strategy should be followed in real life as well.

Our study has several limitations that should be considered while interpreting our results. First, it is a retrospective study; therefore, causality relations are difficult to assess. Second, another inherent limitation of this kind of study is information bias. Sunitinib use began in February 2009, just months after its approval in Brazil. Therefore, it is reasonable to consider that the AEs of this TKI were more accurately reported by the attending physicians and oncology fellows. In September 2013, when pazopanib began to be used in our institution, the physicians already had experience with this class of medication, and perhaps the AEs were not as well described. Because of that, and in addition to having data from the COMPARZ trial, we did not make statistical comparisons between the AEs of the two medications.

Our study showed that the use of TKI inhibitors, either sunitinib or pazopanib, as first-line treatment of metastatic RCC is effective and feasible in the Brazilian public health system. However, the median OS of our population is considerably lower than the prospective trials that evaluated the same drugs, for several reasons.^9^ In our opinion, it is crucial to extend the access to these TKIs for the Brazilian population and also for patients in other countries with limited access to targeted therapies. Also, it is imperative to provide sequential systemic treatment options for this population as an attempt to improve survival and offer the best outcomes for patients with metastatic RCC.

## Figures and Tables

**Table A1:**
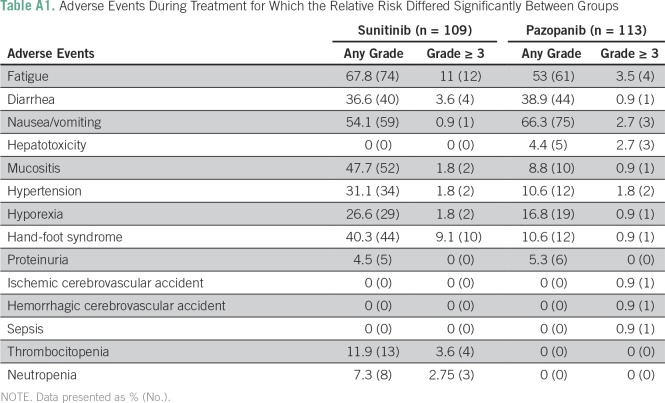
Adverse Events During Treatment for Which the Relative Risk Differed Significantly Between Groups

**Table A2:**
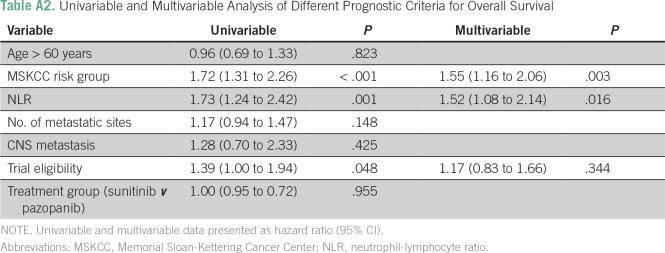
Univariable and Multivariable Analysis of Different Prognostic Criteria for Overall Survival
